# From fragmented innovation to an integrated plant-to-product framework: integrated digital twins and artificial intelligence approaches in phytomedicine

**DOI:** 10.3389/fphar.2026.1876553

**Published:** 2026-07-08

**Authors:** Farhan Amin, Mozaniel Santana De Oliveira, Adnan Amin

**Affiliations:** 1 School of Computer Science and Engineering, Yeungnam University, Gyeongsan, Republic of Korea; 2 Laboratory of Pharmacology of Inflammation and Behavior, Graduate Program in Pharmaceutical Sciences, Institute of Health Sciences, Federal University of Pará, Belém, Brazil; 3 Department of Life Sciences, Yeungnam University, Gyeongsan, Republic of Korea

**Keywords:** artificial intelligence, chemometrics, digital twins, phytochemistry, plant

## Abstract

Even in today’s scientifically advanced era, phytomedicine still faces challenges regarding raw material authentication, chemical variability, process control, and batch variations. Although robust innovations in artificial intelligence (AI) and digital twins (DTs) are being applied to several medicinal fields, their practical implications in phytomedicine remain fragmented. Researchers are employing AI for the identification of plants, drug likeness, quality predictions, and chemometric analysis, while DTs are used in pharmaceutical manufacturing and controlled environmental agriculture. Therefore, this perspective contends for an integrated AI-assisted plant-to-product-based DT framework, connecting genotypes, medicinal plant cultivation, metabolomic, and chemometric profiling to extraction and final product design. A consistent execution of a planned and well-organized framework warrants authentic raw material alongside a validated analytical procedure, standardized metadata, and robust model validation.

## 1 Introduction

Phytomedicine is entering a phase in which its long-standing problems of variability, authentication, and process reproducibility can no longer be adequately addressed by isolated analytical measurements or empirical process adjustments (Ichim and Booker, 2021). Artificial intelligence (AI) and digital twins (DTs) are being used across concurrent disciplines to improve prediction, process control, and decision support (Tegtmeier et al., 2023). Yet, the evidence in phytomedicine remains distributed across parallel, rather than integrated, domains (Sharma et al., 2025). A detailed literature review on the use of AI in medicinal plant research confirms its use as identification tools (Hajam et al., 2024), phytochemical profiling, quality prediction, and detailed chemometric classification on a wider scale. However, DTs, instead of being used in end-to-end phytomedicine systems, are mostly employed in drug manufacturing or in controlled-environment plant production (Chen et al., 2020; Slob and Hurst, 2022).

This asymmetry matters since the manufacturing of herbal medicinal products is not a single-stage manufacturing process. It is a complicated multiscale process comprising biological and industrial systems, starting from genotypes to cultivation, extraction to development of the final therapeutic product (Chen et al., 2023; Lindenmaier et al., 2025). In this context, merging the field of DTs is of prime importance because it offers a dynamic virtual presentation of physical reality, supporting dynamic predictive interventions and assimilation of sensor data and behavior of the models developed (Maharjan et al., 2025). However, the question of the relevance of DTs in phytomedicine is of less value than the central issue of whether the DT field has built a high-quality database, developed model fidelity, and accumulated sufficient regulatory marks to make them scientifically credible and operationally useful.

As this framework is intended for phytomedicine, each DT application must begin with a defined traditional-use context. It should include the accepted botanical name, plant part, preparation method, route of administration, traditional indication, cultural or medical system, and safety information. Such documentation is essential because ethnopharmacological research requires reproducible reporting of local or historical medicinal use (Heinrich et al., 2018). Within the proposed framework, traditional use must guide the choice of plant material, extraction method, bioassay, product form, and validation endpoint.

In this perspective, we hypothesize that AI and DTs can improve phytomedicine only when they are applied to defined plant-to-product quality problems rather than used as broad digital labels. The aims were as follows: i) to clarify the distinction among digital databases, AI prediction tools, digital shadows, cyber–physical systems, and true bidirectional DTs; ii) to evaluate their readiness for botanical authentication, metabolite-based quality prediction, cultivation monitoring, extraction control, formulation optimization, and batch reproducibility; and iii) to propose a bound AI-assisted DT framework linking authenticated plant material, metabolomic profiling, process control, final product release, and regulatory traceability. Such focus is justified by persistent challenges in herbal authentication, chemical variability, adulteration, and reproducible quality control.

## 2 From digital databases to digital twins in phytomedicine

The digitalization of phytomedicine is to be viewed as a maturity continuum rather than a single category. A static digital model is a computational or mathematical representation of a plant, extraction process, formulation, or product, but it is not automatically updated by real-time data (Grieves and Vickers, 2017; Jones et al., 2020). A digital shadow receives data from the physical system, such as cultivation sensors, analytical instruments, or manufacturing equipment, but the information flow is mainly one-directional and does not directly control the physical process (Jones et al., 2020). A cyber–physical system integrates sensing, computation, communication, and control with the physical process, enabling real-time monitoring and coordinated operation (Lee et al., 2015). In contrast, a fully functional digital twin requires a persistent connection between the physical and virtual systems, bidirectional data exchange, predictive modeling, and feedback-driven intervention, allowing the digital network to update its state and support corrective or optimized actions of the physical system (Grieves and Vickers, 2017; Semeraro et al., 2021). Therefore, phytomedical platforms that only store chemical data or classify samples using AI may not be described as DTs unless they include real-time updating, predictive simulation, and closed-loop decision support (Table 1). Various databases, AI-assisted chemometric models, digital shadows, and true DT systems differ substantially in readiness, scalability, and reproducibility requirements. Table 2 critically compares their operational maturity.

**TABLE 1:** AI-enabled and digital twin-enabling platforms relevant to phytomedicine quality, authentication, and plant-to-product integration.

Platform	Digital type	Main phytomedicine application	Reported outcome	Key limitation	References
TCMSP	Systems pharmacology database	Herb–metabolite–target–disease mapping	Supports screening of active metabolite, targets, and pharmacokinetic properties for herbal medicines	Data coverage and target predictions require experimental validation	Ru et al. (2014)
BATMAN-TCM/BATMAN-TCM 2.0	Bioinformatics/target-prediction platform	Ingredient–target prediction and pathway analysis	BATMAN-TCM 2.0 expanded known and predicted traditional Chinese medicine ingredient–target interactions	Mechanistic predictions remain hypothesis-generating	Kong et al. (2023)
ETCM/ETCM 2.0	Curated TCM knowledge platform	Herbs, formulas, ingredients, targets, diseases, and quality-marker exploration	Provides standardized data for mechanism investigation, drug discovery, and quality-marker identification	Not a validated manufacturing or quality-control DT system	Zhang et al. (2023)
HERB	Pharmacotranscriptomic database	Herb/ingredient–gene–disease association mining	Integrates high-throughput and reference-guided data for TCM mechanism studies	Requires biological validation of predicted associations	Fang et al. (2021)
IMPPAT	Phytochemical database	Indian medicinal plant–phytochemical–therapeutic use mapping	Curates medicinal plants, phytochemicals, and therapeutic-use associations	Does not establish efficacy, safety, or batch quality	Mohanraj et al. (2018)
TCMBank	AI-assisted TCM database	Herb–ingredient–target–disease text mining and lead discovery	Links herbal medicines, chemical ingredients, targets, and diseases using intelligent text mining	Literature-derived associations may contain bias or incomplete annotation	Lv et al. (2023)
DCABM-TCM	Bioavailable-constituent database	Blood-absorbed constituents and metabolites of TCM	Supports mechanism analysis using experimentally detected blood constituents	Limited to reported absorbed metabolites and available pharmacokinetic evidence	Liu X. et al. (2023)
Chemometric AI models	Machine learning/multivariate analytics	Authentication, adulteration detection, origin classification, and quality grading	Supports discrimination of herbal species, origins, and chemical-quality classes	Requires authenticated references materials and external validation	Gad et al. (2012)
Digital twin for extraction process design	Process digital twin	Natural-product extraction modeling and process optimization	Provides model-based support for extraction design, operation, and lifecycle analysis	Mostly unit-operation focused; upstream plant variability remains weakly integrated	Uhlenbrock et al. (2020)
Greenhouse/plant-factory DTs	Cultivation digital twin	Controlled-environment cultivation, monitoring, and optimization	Supports greenhouse or plant-factory data integration and operational optimization	Often models growth or equipment performance rather than bioactive metabolite trajectories	Slob and Hurst (2022)

**TABLE 2:** Critical comparison of digital technology classes for plant-to-product digital twins in phytomedicine.

Technology class	Digital maturity	Readiness	Key limitation	Reproducibility need	Role in the DT framework	References
Phytochemical databases	Static knowledge base	High for retrieval	No real-time control	Curated and updated annotations	Knowledge layer	Klein-Júnior et al. (2021)
AI-assisted chemometrics	Predictive AI tool	Moderate for QC	Dataset bias	External validation	Quality-prediction layer	Noviana et al. (2022)
Controlled cultivation systems	Digital shadow/partial DT	Moderate	Often biomass-focused	Sensor and metabolite validation	Cultivation-data layer	Verdouw et al. (2021)
PAT/QbD manufacturing	Cyber–physical control	High in pharma	Needs validated analytics	CPP–CQA validation	Process-control layer	Amidon et al. (2014)
Process DT models	Partial/operational DT	Moderate	Unit-operation focus	Prospective process testing	Manufacturing optimization	Phalak et al. (2023)
End-to-end plant-to-product DT	Fully bidirectional DT	Early-stage	High interoperability burden	Multi-site prospective validation	Integrated decision-support layer	Ferko et al. (2022)

Table 1 should be interpreted as an inventory of enabling resources, not as evidence of validated pharmacological effects. Systems pharmacology databases and target prediction platforms are useful for hypothesis generation, but their outputs may include literature bias, incomplete annotation, and false-positive target recognition (Chen et al., 2006). Therefore, database-derived targets, pathways, or activity claims must not be employed as validated DT inputs unless supported by experimental, pharmacological, or clinical evidence (Dong et al., 2021).

## 3 Evidence and implementation readiness

### 3.1 AI-assisted authentication and chemometric quality prediction

This area of phytomedicine is where AI outperforms the implementation of DT platforms. As discussed, AI methods are already being applied to image-based recognition of herbs (plant identification), feature extraction, chemometrics, and metabolomics-assisted profiling (Banerjee et al., 2025; Ravjot et al., 2025). These trends suggest that instantaneous implementations of digital phytomedicine may not be solely driven by DTs; instead, they may depend on AI-augmented DTs, where machine learning models help infer latent quality states. A major challenge in herbal medicine is sample authentication (plant batches**)** since species substitution, adulteration, mislabeling ingredients, and inconsistencies in batch authentication are common (Ichim et al., 2020; Ichim and Booker, 2021). Although orthogonal authentication measures, including DNA-based methods, chemical profiling, microscopy, and bioactivity-based assays, are used, these are laborious, expensive, and time-consuming (Woodley et al., 2021; Raclariu-Manolică et al., 2023). Consequently, the path forward is to use DT-based systems to integrate rigorously validated measurements across the cultivation-to-manufacturing process.

### 3.2 Cultivation of digital shadows and metabolite-aware monitoring

Contrary to this trend, the literature on plants is less directly aligned with herbal medicine quality, despite its prominence. DT-based investigations in agricultural and plant-cultivation factories focus on the architecture of greenhouses, data coupling, working and efficiency of monitoring equipment, and production logistics (Howard et al., 2020; Liu J. et al., 2023). Indeed, such frameworks offer improved environmental control and operational efficiency, but they rarely model the trajectory of secondary metabolites with bioactivity within phytomedicine (Sharma et al., 2025). Such a key gap has to be considered since the therapeutic value of medicinal herbs mainly depends on the chemical structure and bioeffects of secondary metabolites, rather than on biomass alone (Elshafie et al., 2023). Sufficient scientific evidence supports the fact that using plant bioprocess standardization measures (plant extracts) and metabolomics-guided cultivation can stabilize metabolite output. However, these advances are not yet routinely embedded in DT architectures (Marchev et al., 2020).

### 3.3 PAT/QbD-enabled extraction and manufacturing models

The strongest case for DT adoption in phytomedicine is derived from the pharmaceutical process-associated literature. Reviews and implementation studies show that DTs can be coupled with quality by design (QbD) and process analytical technology (PAT) to support real-time monitoring, process-state estimation, control of critical quality attributes, and lifecycle optimization (Phalak et al., 2023; Juckers et al., 2024). Such evidence is especially relevant since at least one study on natural products has already extended the use of DT logic in designing extraction processes and building their operational capacity. This observation provides an argument for the implications of validated process models as operational DTs in regulated phyto-pharmaceutical settings (Uhlenbrock et al., 2020). In other words, the downstream part of a DT-based phytomedicine pipeline is no longer speculative; this is further evident from another recent investigation involving the development of an autonomous hydrodistillation system for volatile phytochemicals by integrating DTs**,** PAT**,** and advanced process control. It was evident that DT-enabled process control markedly reduced the ecological footprint of extraction by **≤**46.5% and extraction costs by 22.4% (Uhl et al., 2024)**.**

### 3.4 Toward an AI-assisted plant-to-product digital twin framework

The most useful near-term target in the abstract, therefore, is not an overextended “digital twin of phytomedicine.” It is a plant-to-product digital twin with a clearly bounded scope. Such a framework would connect the following: **i)** cultivation-related variables that shape plant physiology, **ii)** metabolomic or chemometric signatures that report phytochemical state, **iii)** extraction and purification parameters that determine the transfer and enrichment of bioactive constituents, and **iv)** release specifications that define the final product. Such a design would also align with evidence clusters available in the literature: mature DT models for pharmaceutical processing, developing DT models for controlled-environment-based agriculture, robust AI models for herbal recognition and quality prediction, and a substantial body of work on herbal authentication and standardization (Chen et al., 2020; Slob and Hurst, 2022).

In phytomedicine practice, designing an AI-assisted plant-to-product digital twin may use a layered cyber–physical system. An initial verification of plants’ identity must rely on this input layer, genotype or accession, plant part, developmental stage, cultivation conditions, harvest time, and post-harvest handling because herbal products remain vulnerable to misidentification and adulteration, requiring orthogonal authentication approaches such as DNA-, chemical-, and microscopic-based methods (Ichim and Booker, 2021; Nazar et al., 2025). The sensing layer must integrate environmental, phenotyping, metabolomic, and chemometric upstream data from cultivation systems, consistent with agricultural DT concepts (Pylianidis et al., 2021). These have to be connected to the manufacturing layer, where extraction parameters, marker-metabolite abundance, impurity profiles, and formulation attributes are monitored utilizing validated analytical workflows, PAT, and QbD principles (Hinz, 2006; Uhlenbrock et al., 2020). Furthermore, AI models should support deviation detection, quality-state prediction, process optimization, and controlled model updation using externally verified datasets. Finally, validation must include biological authentication, analytical reproducibility, external AI-model testing, and prospective process verification to ensure regulatory traceability, auditability, and reproducible decision-making (Moons et al., 2025).

A practical plant-to-product DT requires a minimal data model at each layer rather than unrestricted data accumulation. The cultivation layer should define the biological state and environmental relationships of the plant. The metabolomic layer must convert this state into a chemical-quality vector. The extraction layer then has to translate the vector into process parameters and extract attributes. The product layer should link the extract profile to formulation performance, release specifications, and regulatory traceability. Such layered data handoff is consistent with agricultural DT concepts, chemometric quality control, extraction DTs, and QbD-based pharmaceutical development (Uhlenbrock et al., 2020; Pylianidis et al., 2021).

Thus, data must flow bidirectionally from authenticated plant-derived inputs and cultivation-associated sensors to metabolomics, extraction control, formulation assessment, and final product release (Nazar et al., 2025). The defined impact can be enabled by feedback loops if there exists any deviation from validated specifications covering metabolite profiles, extraction efficiency, or critical quality-associated attributes.

Uncertain metabolite annotation should be treated as an acknowledged limitation of the DT state layer. In LC-MS-based metabolomics, many detectable features may remain unannotated, and misannotation can distort biointerpretation (Chaleckis et al., 2019). Therefore, DT cannot treat all metabolomic features as equally reliable, which must be assigned annotation-confidence levels, separating confirmed from putatively annotated metabolites and unknown spectral features. The latter may still support batch discrimination or quality prediction but should not be utilized for mechanistic claims unless validated by reference standards, MS/MS matching, retention time, or orthogonal evidence (Broeckling et al., 2016). The DT must also record uncertainty scores and update annotations as spectral libraries and reference datasets keep improving.

As an illustrative use case, the framework can be applied to *Centella asiatica* (L.) Urb., which contains triterpenoids such as asiaticoside and madecassoside (Biswas et al., 2021). This is a proposed example and does not imply the existence of a validated end-to-end DT for this species. The cultivation layer records accession, plant part, growth stage, cultivation conditions, harvest time, and drying method. The metabolomics layer quantifies marker triterpenoids via HPLC or LC-MS (Xing et al., 2009). The extraction layer models the solvent, time, temperature, yield, and marker transfer. The product layer links extract composition to formulation stability, release specifications, and batch documentation. Validation requires authenticated plant material, confirmed analytical methods, independent batch testing, relevant bioassays, and final release testing.

### 3.5 From fragmented tools to validated systems

The distinction between various levels of digital maturity in phytomedicine is critical. Traditional process control remains the most regulatory-familiar approach because it relies on pre-defined critical quality attributes, validated analytical methods, and controlled manufacturing parameters; however, its capacity to capture upstream biological variability in medicinal plants is limited (Yu et al., 2014; Park et al., 2022). AI-assisted chemometrics can improve authentication, adulteration detection, origin classification, and quality prediction, particularly when combined with spectroscopic or chromatographic fingerprints, but its reproducibility depends on characteristic datasets, validated methods, and suitable reference materials (Abdul Rohman et al., 2019; Hosbas Coskun et al., 2021). Digital shadow systems improve real-time process visibility by transferring data from the physical process to the digital model, whereas true DT systems require bidirectional data exchange, predictive modeling, and feedback-driven intervention, rendering them more powerful but also more difficult to validate and scale (Kritzinger et al., 2018). Thus, phytomedicine implementation may prioritize proven digital shadows and AI-assisted quality prediction as transitional steps toward fully bidirectional DT systems.

The next stage of digital phytomedicine must prioritize validation rather than technological expansion alone. First, cultivation-based studies should move from generic environmental monitoring to metabolite-aware modeling that links genotype, developmental stage, and stress exposure with therapeutic chemistry (Li et al., 2020; Pylianidis et al., 2021). Second, extraction and formulation DTs must incorporate upstream plant-quality data instead of being limited to isolated unit operations (Yi et al., 2021). Third, AI models for classification or quality prediction have to be trained on analytically authenticated materials and have to be tested across batches, seasons, geographies, and supply chains. Fourth, regulatory discussion should focus on whether these systems are transparent, reproducible, auditable, and compatible with QbD framework principles (Lindenmaier et al., 2025).

### 3.6 Validation hierarchy and regulatory implementation pathway

DT **validation for** phytomedicine needs a careful framework before practical applications, using a series of measures. In the first instance, a thorough authentication of biological input through genetic, plant-, or phytochemistry-based evidence is needed (Nazar et al., 2025). Second, the analytical workflows employed must be assessed for sensitivity, reproducibility, and batch–batch comparisons (Hinz, 2006). The third vital step involves performance verification of developed AI models through external datasets—seasonal variations, diverse geographic origins, plant parts, and processing histories (Moons et al., 2025). The final step is a prospective approval of process models under actual production conditions (Bitencourt et al., 2024). Without such hierarchy, a DT may remain a descriptive model rather than a reliable decision-support system.

A DT-predicted “equivalent product” should not be accepted based only on chemical fingerprint similarity; these support batch consistency but do not prove therapeutic equivalence (Noviana et al., 2022). Herbal efficacy may also depend on active markers, synergistic constituents, bioavailability, metabolism, and matrix effects (Awang, 2004). Therefore, DT-based likeness must require chemical similarity, along with biopharmaceutical testing, mechanism-relevant bioassays, or pharmacodynamic/clinical evidence where needed (Loew et al., 2002).

The proposed DT framework should align with the ConPhyMP guidelines, which provide a consensus standard for reporting medicinal plant materials, extract preparation, and phytochemical characterization (Heinrich et al., 2022). In this context, it can define the minimum chemical metadata required before an extract enters the DT pipeline. Such information must include plant identity, part, collection or cultivation details, extraction method, extract type, analytical platform, marker metabolites, and the full chemical profile. Without this extent of reporting, DT predictions may be irreproducible or non-comparable across laboratories, batches, or studies.

**FIGURE 1:**
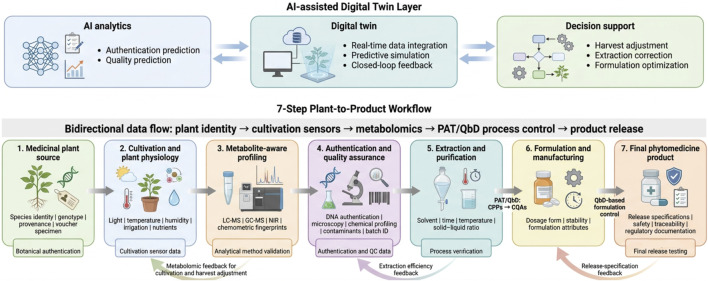
Conceptual architecture of an AI-assisted plant-to-product digital twin framework for phytomedicine.

Regulatory implementation must be anchored in GACP, GMP, and data-integrity principles. Upstream cultivation and collection have to follow GACP to ensure traceable and authenticated botanical material. Downstream processing should align with GMP expectations for validated computerized systems, access and change control, audit trails, and data retention (Chan, 2003). Analytical reproducibility should be supported by validated methods and lifecycle control (Chikezie and Ojiako, 2015). Furthermore, AI modules must have a predefined context of use, documented data provenance, version control, explainability, performance monitoring, and human oversight (Khalid et al., 2016). Therefore, a phytomedicine DT should generate audit-ready records that connect plant authentication with decisions on final product release. This framework has to complement the existing herbal medicine regulations and not replace them. Botanical-derived drug guidance by the FDA, EMA/HMPC requirements, and China’s NMPA framework emphasize raw-material control, manufacturing consistency, defined specifications, and quality documentation. Regulatory acceptance may, therefore, be most realistic, first at the **digital model** level for development and risk assessment, as well as the **digital shadow** level for traceability, PAT monitoring, and audit-ready records (Figure 1).

The framework has to be implemented as a staged maturity pathway, but not as a mandatory full-DT requirement. For small-to-medium producers, the initial realistic step is a minimally **viable digital shadow**, which may include authenticated raw material records, voucher documentation, batch IDs, basic cultivation or collection logs, low-cost microscopy or HPTLC/HPLC testing, and digital batch records. More advanced techniques, such as metabolomics, PAT, and predictive DT modeling, may be introduced through shared analytical facilities, academic partnerships, or regional quality-control hubs. This proportional approach is vital as limited infrastructure, expertise, and regulatory capacity remain major barriers in many herbal medicine settings (Castro-Dionicio et al., 2025). Thus, the framework should be viewed as a scalable pathway toward digital quality assurance, rather than an immediate technological threshold.

## 4 Conclusion

Current evidence indicates that fully validated, bidirectional twin digital systems for end-to-end phytomedicine remain at an early stage; however, several enabling metabolites are available. These include AI-assisted authentication, chemometry-based quality prediction, metabolite-aware cultivation, and DT-based extraction or manufacturing models. Instead, the availability of more measured interpretations in the form of several robust technologies is needed to develop such systems (DTs). Future technological advancements in phytomedicine are thus expected to use DTs as a broad label. As an alternative, validated, multiscale, AI-assisted frameworks can be of great importance in connecting medicinal plant biology with phytopharmaceutical control. All such futuristic modules can provide sufficient capacity.

## Data Availability

The original contributions presented in the study are included in the article/supplementary material; further inquiries can be directed to the corresponding author.
